# Cell-Free Protein Synthesis: Chassis toward the Minimal Cell

**DOI:** 10.3390/cells8040315

**Published:** 2019-04-05

**Authors:** Ke Yue, Yiyong Zhu, Lei Kai

**Affiliations:** 1 The Key Laboratory of Biotechnology for Medicinal Plants of Jiangsu Province, School of Life Sciences, Jiangsu Normal University, Shanghai Road 101, Xuzhou 221116, China; kyue@jsnu.edu.cn; 2 Jiangsu Provincial Key Lab for Organic Solid Waste Utilization, National Engineering Research Center for Organic-based Fertilizers, Jiangsu Collaborative Innovation Center for Solid Organic Waste Resource Utilization, Nanjing Agricultural University, Nanjing 210095, China; yiyong1973@njau.edu.cn; 3 Department of Cellular and Molecular Biophysics, Max Planck Institute of Biochemistry, D-82152 Martinsried, Germany

**Keywords:** cell-free protein synthesis, chassis, gene circuits, micro-compartments, stochasticity

## Abstract

The quest for a minimal cell not only sheds light on the fundamental principles of life but also brings great advances in related applied fields such as general biotechnology. Minimal cell projects came from the study of a plausible route to the origin of life. Later on, research extended and also referred to the construction of artificial cells, or even more broadly, as in vitro synthetic biology. The cell-free protein synthesis (CFPS) techniques harness the central cellular activity of transcription/translation in an open environment, providing the framework for multiple cellular processes assembling. Therefore, CFPS systems have become the first choice in the construction of the minimal cell. In this review, we focus on the recent advances in the quantitative analysis of CFPS and on its advantage for addressing the bottom-up assembly of a minimal cell and illustrate the importance of systemic chassis behavior, such as stochasticity under a compartmentalized micro-environment.

## 1. Introduction

Fascinated by the emergence of life from non-living matter through billions of years of evolution, scientists began to comprehend and reconstruct how this occurred. Advances in multi-disciplinary research fields, crossing physics, chemistry, and biology, allowed us to explore the essence of life via experimental approaches. Although, until today, the definition of the full characteristics of life and what are essentials for a minimal cell are debated, though a number of features have been agreed upon, i.e., regarding compartmentalization, metabolism (energy and mass exchange), self-organization, growth and division, adaptability and mobility, and information processing [1,2,3,4]. Those processes have become target functions to be reconstructed using in vitro modular systems and have integrated stepwise toward the minimal cellular mimicry. From this point of view, aiming at understanding the essence of life, minimal cell projects seek to reconstitute cellular processes controllably and predictably via a minimum set of compounds [1,5]. When designing and building such a minimal biological system, one can proceed either ‘top-down’ or ‘bottom-up’ [6]. In a top-down approach, a bacterial target genome is continuously reduced to a minimal gene set in vivo; a bottom-up approach relies on the production and purification of functional molecules, which are then combined in vitro with the goal of assembling a minimal cell [7].

Since its first application in deciphering genetic codes [8], cell-free protein synthesis (CFPS) has emerged as an important recombinant protein production method [9,10,11,12,13,14,15,16]. CFPS, being a framework for understanding, harnessing, and expanding biological systems in vitro, has also been used as an important toolbox in other fields of synthetic biology [2,3,17,18]. CFPS, as indicated by its name, refers to the expression of recombinant proteins without living cells. Either cell extracts or individual purified enzymes are used as the machinery for protein transcription/translation [11]. The development of minimal cell projects via a bottom-up approach came along with the quest for pre-biotically plausible routes to the origin of life [19], experimentally repeating the transition from pure chemical compounds to living systems [20]. Hence, the CFPS system as a fully reconstituted system naturally became useful for such projects.

However, we are still far from the ultimate goal of obtaining a minimum cellular system. As mentioned above, full agreement on the essential properties for a minimal cell has not been reached. Although the critical characters of life are still debated, such features after extensive discussion could lead to the design and assembly of minimal cells [21,22]. Moreover, the stepwise construction of a minimal cell could provide new insights into the essence of living systems. Finally, revealing the fundamental principles of a living system will accelerate related applications, certainly in this sense beneficial for biotechnology in general.

**Figure 1:**
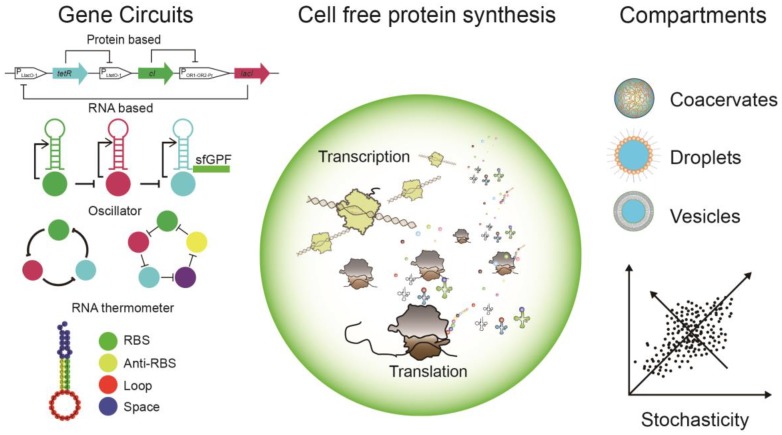
Cell-free protein synthesis (CFPS) within various compartments and its regulation via gene circuits. The CFPS system hosts the core transcription and translation processes, providing the chassis/framework for different cellular mimicry modules/systems. A number of regulatory elements were introduced and validated to manipulate the protein synthesis within CFPS systems. Both RNA and protein-based gene circuits were built to regulate target protein expression on the transcription level via tuning corresponding mRNA concentration. With such design principles, large genetic networks were successfully realized, i.e., 3- and 5-node ring oscillators. On the translational level, RNA thermometers were employed and were able to control translation initiation via tuning the availability of the ribosomal binding sites (RBS). Different materials were applied for creating the physical boundary to encapsulate the CFPS reactions, including coacervates, water in oil droplets, and lipid vesicles. System stochasticity starts to influence the output of gene expression when CFPS reactions were encapsulated. RBS: ribosomal binding site; Anti-RBS: anti-ribosomal binding site; sfGFP: super folder green fluorescent protein; *tetR:* gene sequence coding Tet Repressor proteins; *cl*: gene sequence coding cl protein that binds *OR1* and *OR2* sites within *P_R_* promoter; *lacI:* gene coding lac repressor; *P_LlacO-1_*, *P_LtetO-1_*, and *P_OR1-OR2-Pr_*: promoter sequences that can be regulated via corresponding repressor proteins.

As shown in Figure 1, in this review, we set our focus on the characterization of a CFPS system, in particular about the regulation of protein expression via genetic circuits and designed micro-compartments, as well as the principles to reconstitute biological patterns, moving toward a self-organizing system. Additionally, systematic stochasticity, molecular crowding effects, and their important roles in a stepwise assembly of the multi-functional minimal system are briefly discussed. Finally, we point out the trend toward the quantitative analysis of CFPS systems, which will be beneficial to the integration and hierarchical assembly of a minimal cellular system in vitro.

## 2. Regulation of the CFPS System

### 2.1. Genetic Circuits

Cells develop a set of regulatory tools to sense and process stimuli (information) from the external environment and internal physiological states [23]. In response to constant environmental changes, cellular activities are tuned through a set of regulatory elements, controlling various gene expressions. Such a regulatory system is encoded within genetic networks, interconnected webs of regulatory molecules, synchronizing gene expression in defined patterns, namely ‘gene circuits’ [24]. Similar to electrical circuits, gene circuits are analogies abstracted from well-characterized genes and gene products that respond to a stimuli signal [25,26]. Since the pioneering work from Elowitz, Leibler, and Gardner et al., the single cell system has been conceived as the framework which was composed of standard interaction circuits capable of receiving input signals, executing a serial logical computation, and producing output signals [27]. Therefore, most known gene circuits using such a cell system were discovered via introducing genetic or phrenological perturbation of the model system via a top-down approach [28]. The discovery of such gene circuits did not necessarily give a clear answer on the design and selection principles for a particular functional gene circuit unit [29]. The initial goal of such a synthetic approach was to create autonomous genetic circuits, functioning independently from endogenous cellular circuitry, and finally replacing the endogenous circuitry completely [29]. In addition, continuous efforts in developing computational tools greatly accelerated the characterization and design of genetic regulators [23], which resulted in a number of well-characterized regulatory elements and design principles. However, generic limitations of in vivo modular systems greatly hamper the designing of new circuits, so a limited set of molecules can be successfully implemented—far less than those contained in the simplest organisms [23,24]. The chassis behavior of a cell system requires high compatibility with the existing regulatory elements, often resulting in an unpredictable output; on the other hand, the implementation of new regulatory gene circuits into cells often requires a long procedure until the output signal can be characterized. In addressing the above challenges, a complementary in vitro approach was employed and developed, offering a more flexible chassis as a simplified cell mimicry environment for characterization of the output of designed gene circuits [30,31,32,33,34]. Different in vitro systems were applied for testing designed gene circuits, including nucleic acid systems, hybrid systems and transcription, and translation systems (we direct our readers to a detailed review [35]). Such a complementary in vitro approach, namely the bottom-up approach in synthetic biology, followed by the design–build–test workflow, helps to reveal the fundamental regulatory mechanism and is devoid of the influence of cellular chassis behavior. Next, we focus on the gene circuits investigated in the in vitro transcription and translation system, also referred to as a CFPS or Cell-free TX-TL (Transcription-Translation) system.

### 2.2. Protein Based Gene Circuits

Since the first gene circuit in the CFPS system was established by Noireaux, Bar-Ziv and Libchaber in 2003 [30], a broad range of genetic circuits have been characterized and championed by different groups (see Table 1 for details). Early CFPS systems, especially the T7 polymerase-based system, employed phage polymerases as a strong and efficient transcriptional machinery to provide a sufficient amount of mRNA for translation [36]. However, due to the highly efficient phage polymerase-based transcription machinery, a limited number of regulatory elements could be used, which hampered the design of large and complex gene circuits. In addressing such a challenge, the research group of Noireaux used the endogenous RNA polymerase from *E. coli* instead of T7 polymerase to support transcription in the CFPS system. Due to the well-studied transcriptional control elements in *E. coli*, a variety of control elements, i.e., sigma factor-based regulators, could be used in such a CFPS system. After extensive and comprehensive characterization in the CFPS system, the transcription repertoire of the CFPS system based on endogenous *E. coli* RNA polymerase was greatly extended, resulting in a large number of transcription-regulatory factors [37,38]. Based on the above-verified transcription factors in the CFPS system, modular circuit motifs, such as the logical AND gate, multiple stage cascades, negative feedback loops, positive feedback loops, RNA transcriptional cascades with a protein regulated incoherent feed-forward loop, and in vitro ring oscillators, were successfully implemented (see Table 1 for details). Such in vitro gene circuits based on CFPS allow systems to operate in a synthetic environment considerably more simply than do in vivo model systems, though the two systems are functionally similar. Furthermore, the rapid circuit design–build–test workflow allows one to probe fundamental aspects of gene circuit operation which are otherwise masked by the complex cellular environment in vivo [39].

### 2.3. RNA-Based Gene Circuits

Not only restricted to proteins, regulatory molecules can also consist of RNAs. Takahashi, et al. successfully established an RNA transcriptional cascade via RNA transcriptional attenuators (pT181 and its mutants) as the central regulator, performing as a transcriptional on/off switch [42]. Beyond the regulatory effect on the transcriptional level, RNAs can also regulate gene expression on a translation level. Classical regulation control mainly focused on mutating ribosomal binding sites so as to turn the translation rate via the binding kinetics of ribosomes [45]. Recently, noncoding RNA, such as riboswitch (sRNA and RNA thermometers) [44,46,47], and catalysis (ribozymes) can also act as regulatory elements in relation to the translation to tune the expression of the specific gene [48,49]. For instance, riboswitches, located within the 5′-UTR regions of mRNA, can regulate downstream gene expression in response to ligand binding directly to the mRNA [50,51].

## 3. Programming Spatiotemporal Patterns—Toward the Minimal Cell Division System

Biological systems are highly organized. Even in the simplest prokaryotic cell system, synchronized molecular rearrangement can be found. Besides the regulatory machinery encoded via genetic circuits, biological systems also develop another strategy to organize protein expression on large space and time scales. As first described in the Turing/reaction-diffusion (RD) model, the simple interaction of two components with different diffusion coefficients lead to a spatiotemporal pattern formation under certain theoretical conditions [52]. Such pattern formation exists in different biological systems, from single cellular to animal embryo development processes [53]. One of the well-studied examples of self-organization and pattern formation both in vivo and in vitro was the bacteria MinCDE system [54]. Min proteins constantly oscillate from pole to pole (long axis in vitro) merely via the biochemical properties of MinD and MinE. Upon ATP binding and dimerization, MinD cooperatively binds the membrane via an amphipathic C-terminal membrane targeting sequence (mts) [55]. Both in vivo and in vitro experiments showed that the Min protein oscillation system was able to sense and react to morphological changes via dynamic Min protein patterns. Here, we would like to direct our readers to several comprehensive reviews on this topic [52,53,56]. Such a Min protein oscillation system is key to correctly positioning the contract ring—the ‘Z-ring’—at the mid-cell of *E. coli* [57,58,59]. Encapsulated Min proteins can even act as additional mechanical forces in giant unilamellar vesicles (GUVs), resulting in a rapid deformation of GUVs. This may provide simple autonomous division machinery for lipid vesicles systems [60].

## 4. Toward Self-Organization in CFPS Systems

Moving beyond the Min protein oscillation pattern, how can one reconstitute a reaction-diffusion expression pattern in a CFPS system? What is the prerequisite for such a complex system? Considering the Turing model, a reaction-diffusion model for a chemical signal would be described by differential equation *∂u*⁄*∂t* = *D*∂^2^*u*/∂*x*^2^+*f(u)* for local concentration *u*(*x*, *t*) in spatial coordinate x as a function of time t. In this model, the rate of change, *∂u*⁄*∂t*, is determined by a diffusion operator in space, *D*∂^2^*u*/∂*x*^2^, with a coefficient *D*, and by a local nonlinear reaction function, *f(u)*, which includes sources and degradation terms as well as molecular interactions and feedback regulation [56,61,62]. Thus, all the terms in this equation should be implemented in order to build a self-organization pattern in a CFPS system.

As a closed system, particularly within cell-size compartments, the CFPS system has suffered from the fast decay of protein production due to a loss of enzymatic activities, resource consumption, and product accumulation [62]. This has led to a chemical equilibrium, therefore limiting the complexity and size of the gene network [63]. Different approaches have been implemented in order to overcome this challenge: (1) the passive exchange of substrates via the incorporation of a pore-forming protein complex, i.e., α hemolysin (αHL) [30]; (2) the positive degradation of mRNAs and proteins using RNase and protease to improve the turnover of both mRNAs and proteins (however, only mRNA can be maintained in a steady state, which indicates that an extra mechanism might require the support of a steady translation rate) [40]; (3) periodic dilution of CFPS reactions via a fresh reaction mixture and a DNA template enabling continuous nutrient exchange, leading to steady transcriptional and translational reaction rates [63]; and (4) diffusive DNA compartments based on immobilization of DNA on the surface of circular micro-compartments connected via thin capillaries to a feeding channel of a CFPS reaction mixture [64]. Such a design would allow for a steady state expression via creating source-sink dynamics with a combination of synthesis and degradation [65,66].

Besides an effective turnover mechanism, maintaining a biochemical non-equilibrium is also essential for the construction of a dynamic system, which involves two fundamental principles: feedback and nonlinearity [34]. Feedback is at the center of the network design, which can be constructed with either two mutually inhibitory genes [37] or an autocatalytic gene and its inhibitor [65]. Non-linearity can be introduced by the cooperative binding of regulatory proteins [67], enzymatic degradation [68], and the network topology. In order to regulate gene expression in a heterogeneously distributed cellular environment, controllable diffusion communication should be established. Such sensing and communication designs were first verified through multi-compartment systems. For instance, two amphiphilic inducer molecules *N*-acyl-l-homoserine lactones (AHLs) or isopropyl-β-d-thiogalactopyranoside (IPTG) were investigated as signaling molecules for the communication between water/oil droplets, artificial cells, and *E. coli* [69,70].

## 5. Compartmentalization

As one of the essential properties of life, physical boundaries distinguish living matter from non-living environments [71], which allow for the maintenance of non-equilibrium dynamics. Different materials can be used for the in vitro compartmentalization (IVC) process, starting from liquid–liquid phase separation to biomimetic lipid vesicles (examples shown in Figure 1). The first well-studied IVC process was achieved by simply mixing two immiscible fluids by either agitation or vortexing. For instance, an aqueous solution and oil could form self-assembled emulsion water/oil droplets [72,73], allowing for an aqueous environment for biochemical reactions. Later, biomimetic compartments such as lipid vesicles/liposomes [74,75,76], polymersomes [77,78], and proteinosomes [68,79] were developed for the IVC of various reactive components, ranging from reactions catalyzed by a single enzyme, to multi-step reactions driven by enzyme cascades [80,81], and finally to fully functional transcription and translation systems [30]. One major drawback of conventional methods based on processing bulk samples upon spontaneous self-assembly was the inhomogeneity [82], often resulting in poly-dispersed compartments and low encapsulation efficiency [83,84]. The development of micro-fabrication-based microfluidic devices provides a solution to address the above challenges, which could lead to homogeneous droplets and unilamellar vesicles with greatly improved encapsulation efficiency [85,86].

## 6. Stochasticity

Cellular environments are often different from simplified in vitro environments. For instance, heterogeneity and stochasticity are commonly found in various prokaryotic and eukaryotic cells [87,88,89,90]. However, such properties, assuming a well-stirred bulk environment, were not considered to be significant, because most biochemical reactions are investigated under a diluted and homogenously distributed system in vitro. The research group of Luisi found that the process of macromolecules encapsulated within self-assembled lipid vesicles showed a power-law distribution rather than the expected Poisson distribution due to the solute self-condensing effect [91]. Similar solute self-concentration effects were also observed when encapsulating ribosomes and complete CFPS systems, consisting of more than 80 individual macromolecules (i.e., enzymes, ribosomes, and transfer RNAs) [92]. Besides the encapsulation process, the existence of systematic stochasticity is of great importance for the regulation of transcription and translation within a CFPS system, particularly when encapsulated within micro-compartments supposed to mimic cellular dimensions, which has been less studied. In a typical diluted in vitro environment, system stochasticity is often omitted due to the assumption that, under a well-stirred bulk environment, a low concentration of molecules and the random nature of their collisions can be eliminated, or at least greatly reduced [93]. However, the minimal CFPS system PURE hosts more than 900 reactions and involves the translation process [94], without considering the whole transcription process [95]. Therefore, this intrinsic randomness of the CFPS system cannot be completely ignored in micro-compartments, even under bulk conditions. Via applying the dual reporter model described by Elowitz and co-workers [96], the stochasticity of the CFPS system in terms of expression noise was investigated in microchambers, droplets, and vesicles, and such noise is a non-trivial factor when considering CFPS within compartments for assembly cellular mimicry systems [97,98]. The previous kinetic model of the CFPS system, established using the deterministic model under bulk environments, should be adapted by including a stochastic model when the CFPS system is applied to such micro-compartments.

## 7. Perspectives

The shift from qualitative to quantitative analysis of the CFPS system has greatly increased its applications, especially for the bottom-up assembly of minimal cells. Different experimental approaches have been taken to systematically investigate the kinetics of CFPS systems, which resulted in a set of quantitative mathematical models that describe transcription and translation rates [99]. In addition, instead of using the overall fluorescence of a reporter protein, the performance of ribosomes in terms of the active fraction and the number of synthesizing cycles was determined, which could indicate the productivity of a particular CFPS system on a single ribosome level [100,101]. Such quantitative analysis of the CFPS system will contribute to more accurate prediction, together with the systematic development and establishment of the transcriptional and translational control toolbox, which allows for the construction of large and complex gene circuits. Furthermore, via the smart design and application of microfluidic devices, a steady state of the CFPS system can be achieved, which shows the success of assembling a self-organization CFPS system [63]. A number of modular cellular mimics were successfully achieved, i.e., an energy regeneration hybrid vesicle system [78], multi-enzyme cascades for CO_2_ fixation (CETCH) [102], *de novo* synthesis of lipids [103], and recently even a self-replicating Φ29 virus DNA system [104]. Such advances offer versatile building blocks and allow for the attempt to synchronize multi-functional artificial systems under the common chassis of CFPS systems. Furthermore, the comprehensive studies of the chassis behavior of CFPS systems have allowed for the possibility to move beyond a single functional system and to synchronize the expression rhythm within. In spite of such success, this is just the beginning of the journey toward the ultimate goal of a minimum cell. The same is true for CFPS systems, being simplified cellular mimics, yet our understanding of such a fully reconstituted system is far from complete and systematic. There are still other physical properties that need to be investigated, i.e., systematic stochasticity and the molecular crowding effect on biochemical reactions in general. Thus, we do believe that further systematic investigation on the chassis behavior of CFPS systems will not only reveal the fundamental principles of higher-order cellular regulation but also accelerate the hierarchical assembly of a minimal cell.

## Figures and Tables

**Table 1:** Regulate motifs tested in the CFPS system and their regulatory functions.

Regulate Elements	Regulation Description	Control Level	References
*E. coli* sigma factors	Transcriptional activation units	Transcription	[37]
SsrA-ClpXP	Positive degradation of reporter protein		[31,40,41]
TetR, LacI, AraC, and lambda repressors Cl and Cro	Inducible transcriptional repression and activation; bistable switches; genetic oscillators		[23,33,37]
Pr, Pr1, and Pr2	Provides constant transcription		[39]
pT181 and its derivatives	RNA transcriptional attenuator; antisense RNA mediated transcriptional repressor; three level RNA transcription cascades		[42]
small transcription activating RNAs	Small RNAs that activate the transcription of a specific gene regulated by a terminator (T181, AD1)		[43]
BetI, PhIF and SrpR, TetR, LacI, and QacR	3,4 and 5-node oscillators		[34]
RNA thermometers as well as other rational designed sequences	control the secondary structure of mRNA leading to control of the ribosomal binding rate	Translation	[44]

## References

[1] Schwille P., Spatz J., Landfester K., Bodenschatz E., Herminghaus S., Sourjik V., Erb T., Bastiaens P., Lipowsky R., Hyman A. (2018). MaxSynBio - Avenues towards creating cells from the bottom up. Angew. Chem. Int. Ed. Engl..

[2] Jia H.Y., Heymann M., Bernhard F., Schwille P., Kai L. (2017). Cell-free protein synthesis in micro compartments: Building a minimal cell from biobricks. New Biotechnol..

[3] Caschera F., Noireaux V. (2014). Integration of biological parts toward the synthesis of a minimal cell. Curr. Opin. Chem. Biol..

[4] Yewdall N.A., Mason A.F., van Hest J.C.M. (2018). The hallmarks of living systems: Towards creating artificial cells. Interface Focus.

[5] Luisi P.L., Stano P. (2011). Synthetic Biology: Minimal cell mimicry. Nat. Chem..

[6] Forster A.C., Church G.M. (2006). Towards synthesis of a minimal cell. Mol. Syst. Biol..

[7] Jewett M.C., Forster A.C. (2010). Update on designing and building minimal cells. Curr. Opin. Biotechnol..

[8] Nirenberg M.W., Matthaei J.H. (1961). The dependence of cell-free protein synthesis in E. coli upon naturally occurring or synthetic polyribonucleotides. Proc. Natl. Acad. Sci. USA.

[9] Casteleijn M.G., Urtti A., Sarkhel S. (2013). Expression without boundaries: Cell-free protein synthesis in pharmaceutical research. Int. J. Pharm..

[10] Rues R.B., Dotsch V., Bernhard F. (2016). Co-translational formation and pharmacological characterization of beta1-adrenergic receptor/nanodisc complexes with different lipid environments. Biochim. Biophys. Acta.

[11] Carlson E.D., Gan R., Hodgman C.E., Jewett M.C. (2012). Cell-free protein synthesis: Applications come of age. Biotechnol. Adv..

[12] Zubay G. (1973). In-Vitro Synthesis of Protein in Microbial Systems. Annu. Rev. Genet..

[13] Spirin A.S., Baranov V.I., Ryabova L.A., Ovodov S.Y., Alakhov Y.B. (1988). A continuous cell-free translation system capable of producing polypeptides in high yield. Science.

[14] Schwarz D., Junge F., Durst F., Frolich N., Schneider B., Reckel S., Sobhanifar S., Dotsch V., Bernhard F. (2007). Preparative scale expression of membrane proteins in Escherichia coli-based continuous exchange cell-free systems. Nat. Protoc..

[15] Schwarz D., Dotsch V., Bernhard F. (2008). Production of membrane proteins using cell-free expression systems. Proteomics.

[16] Bernhard F., Tozawa Y. (2013). Cell-free expression--making a mark. Curr. Opin. Struct. Biol..

[17] Jewett M.C., Calhoun K.A., Voloshin A., Wuu J.J., Swartz J.R. (2008). An integrated cell-free metabolic platform for protein production and synthetic biology. Mol. Syst. Biol..

[18] Jewett M.C. (2014). Cell-free synthetic biology special issue. ACS Synth. Biol..

[19] Mansy S.S., Szostak J.W. (2009). Reconstructing the emergence of cellular life through the synthesis of model protocells. Cold Spring Harb. Symp. Quant. Biol..

[20] Szostak J.W., Bartel D.P., Luisi P.L. (2001). Synthesizing life. Nature.

[21] Trifonov E.N. (2011). Vocabulary of definitions of life suggests a definition. J. Biomol. Struct. Dyn..

[22] Sarma R.H. (2012). A conversation on definition of life. J. Biomol. Struct. Dyn..

[23] Takahashi M.K., Hayes C.A., Chappell J., Sun Z.Z., Murray R.M., Noireaux V., Lucks J.B. (2015). Characterizing and prototyping genetic networks with cell-free transcription-translation reactions. Methods.

[24] Werner E. (2007). An introduction to systems biology: Design principles of biological circuits. Nature.

[25] Mcadams H.H., Shapiro L. (1995). Circuit Simulation of Genetic Networks. Science.

[26] McAdams H.H., Arkin A. (2000). Gene regulation: Towards a circuit engineering discipline. Curr. Biol..

[27] Elowitz M.B., Leibler S. (2000). A synthetic oscillatory network of transcriptional regulators. Nature.

[28] Lim W.A. (2010). Designing customized cell signalling circuits. Nat. Rev. Mol. Cell Biol..

[29] Nandagopal N., Elowitz M.B. (2011). Synthetic Biology: Integrated Gene Circuits. Science.

[30] Noireaux V., Bar-Ziv R., Libchaber A. (2003). Principles of cell-free genetic circuit assembly. Proc. Natl. Acad. Sci. USA.

[31] Shin J., Noireaux V. (2010). Efficient cell-free expression with the endogenous E. Coli RNA polymerase and sigma factor 70. J. Biol. Eng..

[32] Chappell J., Jensen K., Freemont P.S. (2013). Validation of an entirely in vitro approach for rapid prototyping of DNA regulatory elements for synthetic biology. Nucleic Acids Res..

[33] Sun Z.Z., Yeung E., Hayes C.A., Noireaux V., Murray R.M. (2014). Linear DNA for Rapid Prototyping of Synthetic Biological Circuits in an Escherichia coli Based TX-TL Cell-Free System. ACS Synth. Biol..

[34] Niederholtmeyer H., Sun Z.Z., Hori Y., Yeung E., Verpoorte A., Murray R.M., Maerkl S.J. (2015). Rapid cell-free forward engineering of novel genetic ring oscillators. Elife.

[35] Hockenberry A.J., Jewett M.C. (2012). Synthetic in vitro circuits. Curr. Opin. Chem. Biol..

[36] Karig D.K., Iyer S., Simpson M.L., Doktycz M.J. (2012). Expression optimization and synthetic gene networks in cell-free systems. Nucleic Acids Res..

[37] Shin J., Noireaux V. (2012). An E. coli cell-free expression toolbox: Application to synthetic gene circuits and artificial cells. ACS Synth. Biol..

[38] Garamella J., Marshall R., Rustad M., Noireaux V. (2016). The All E. coli TX-TL Toolbox 2.0: A Platform for Cell-Free Synthetic Biology. ACS Synth. Biol..

[39] Siegal-Gaskins D., Tuza Z.A., Kim J., Noireaux V., Murray R.M. (2014). Gene Circuit Performance Characterization and Resource Usage in a Cell-Free “Breadboard”. ACS Synth. Biol..

[40] Shin J., Noireaux V. (2010). Study of messenger RNA inactivation and protein degradation in an Escherichia coli cell-free expression system. J. Biol. Eng..

[41] Karzbrun E., Shin J., Bar-Ziv R.H., Noireaux V. (2011). Coarse-Grained Dynamics of Protein Synthesis in a Cell-Free System. Phys. Rev. Lett..

[42] Takahashi M.K., Chappell J., Hayes C.A., Sun Z.Z., Kim J., Singhal V., Spring K.J., Al-Khabouri S., Fall C.P., Noireaux V. (2015). Rapidly Characterizing the Fast Dynamics of RNA Genetic Circuitry with Cell-Free Transcription Translation (TX-TL) Systems. ACS Synth. Biol..

[43] Chappell J., Takahashi M.K., Lucks J.B. (2015). Creating small transcription activating RNAs. Nat. Chem. Biol..

[44] Sadler F.W., Dodevski I., Sarkar C.A. (2018). RNA Thermometers for the PURExpress System. ACS Synth. Biol..

[45] Chizzolini F., Forlin M., Cecchi D., Mansy S.S. (2014). Gene position more strongly influences cell-free protein expression from operons than T7 transcriptional promoter strength. ACS Synth. Biol..

[46] Ogawa A. (2011). Rational design of artificial riboswitches based on ligand-dependent modulation of internal ribosome entry in wheat germ extract and their applications as label-free biosensors. RNA.

[47] DebRoy S., Gebbie M., Ramesh A., Goodson J.R., Cruz M.R., van Hoof A., Winkler W.C., Garsin D.A. (2014). A riboswitch-containing sRNA controls gene expression by sequestration of a response regulator. Science.

[48] Wieland M., Hartig J.S. (2008). Artificial riboswitches: Synthetic mRNA-based regulators of gene expression. ChemBioChem.

[49] Weigand J.E., Suess B. (2009). Aptamers and riboswitches: Perspectives in biotechnology. Appl. Microbiol. Biotechnol..

[50] Winkler W.C. (2005). Riboswitches and the role of noncoding RNAs in bacterial metabolic control. Curr. Opin. Chem. Biol..

[51] Martini L., Mansy S.S. (2011). Cell-like systems with riboswitch controlled gene expression. Chem. Commun..

[52] Kondo S., Miura T. (2010). Reaction-Diffusion Model as a Framework for Understanding Biological Pattern Formation. Science.

[53] Kretschmer S., Schwille P. (2016). Pattern formation on membranes and its role in bacterial cell division. Curr. Opin. Cell Biol..

[54] Meinhardt H., de Boer P.A.J. (2001). Pattern formation in Escherichia coli: A model for the pole-to-pole oscillations of Min proteins and the localization of the division site. Proc. Natl. Acad. Sci. USA.

[55] Loose M., Fischer-Friedrich E., Ries J., Kruse K., Schwille P. (2008). Spatial regulators for bacterial cell division self-organize into surface waves in vitro. Science.

[56] Cross M.C., Hohenberg P.C. (1993). Pattern-Formation Outside of Equilibrium. Rev. Mod. Phys..

[57] Wu L.J., Errington J. (2012). Nucleoid occlusion and bacterial cell division. Nat. Rev. Microbiol..

[58] Hu Z.L., Mukherjee A., Pichoff S., Lutkenhaus J. (1999). The MinC component of the division site selection system in Escherichia coli interacts with FtsZ to prevent polymerization. Proc. Natl. Acad. Sci. USA.

[59] Osawa M., Anderson D.E., Erickson H.P. (2008). Reconstitution of contractile FtsZ rings in liposomes. Science.

[60] Litschel T., Ramm B., Maas R., Heymann M., Schwille P. (2018). Beating Vesicles: Encapsulated Protein Oscillations Cause Dynamic Membrane Deformations. Angew. Chem. Int. Edit..

[61] Keener J.P., Sneyd J. (1998). Mathematical Physiology.

[62] Tayar A.M., Daube S.S., Bar-Ziv R.H. (2017). Progress in programming spatiotemporal patterns and machine-assembly in cell-free protein expression systems. Curr. Opin. Chem. Biol..

[63] Niederholtmeyer H., Stepanova V., Maerkl S.J. (2013). Implementation of cell-free biological networks at steady state. Proc. Natl. Acad. Sci. USA.

[64] Karzbrun E., Tayar A.M., Noireaux V., Bar-Ziv R.H. (2014). Programmable on-chip DNA compartments as artificial cells. Science.

[65] Tayar A.M., Karzbrun E., Noireaux V., Bar-Ziv R.H. (2015). Propagating gene expression fronts in a one-dimensional coupled system of artificial cells. Nat. Phys..

[66] Tayar A.M., Karzbrun E., Noireaux V., Bar-Ziv R.H. (2017). Synchrony and pattern formation of coupled genetic oscillators on a chip of artificial cells. Proc. Natl. Acad. Sci. USA.

[67] Alon U. (2006). An Introduction to Systems Biology: Design Principles of Biological Circuits.

[68] Huang X., Patil A.J., Li M., Mann S. (2014). Design and Construction of Higher-Order Structure and Function in Proteinosome-Based Protocells. J. Am. Chem. Soc..

[69] Lentini R., Santero S.P., Chizzolini F., Cecchi D., Fontana J., Marchioretto M., Del Bianco C., Terrell J.L., Spencer A.C., Martini L. (2014). Integrating artificial with natural cells to translate chemical messages that direct E-coli behaviour. Nat. Commun..

[70] Weitz M., Muckl A., Kapsner K., Berg R., Meyer A., Simmel F.C. (2014). Communication and Computation by Bacteria Compartmentalized within Microemulsion Droplets. J. Am. Chem. Soc..

[71] Shapiro R. (2007). A simpler origin for life. Sci. Am..

[72] Tawfik D.S., Griffiths A.D. (1998). Man-made cell-like compartments for molecular evolution. Nat. Biotechnol..

[73] Griffiths A.D., Tawfik D.S. (2003). Directed evolution of an extremely fast phosphotriesterase by in vitro compartmentalization. EMBO J..

[74] Schmidli P.K., Schurtenberger P., Luisi P.L. (1991). Liposome-Mediated Enzymatic-Synthesis of Phosphatidylcholine as an Approach to Self-Replicating Liposomes. J. Am. Chem. Soc..

[75] Luisi P.L., Walde P., Oberholzer T. (1999). Lipid vesicles as possible intermediates in the origin of life. Curr. Opin. Colloid Interface Sci..

[76] Chen I.A., Walde P. (2010). From self-assembled vesicles to protocells. Cold Spring Harb. Perspect. Biol..

[77] Discher D.E., Eisenberg A. (2002). Polymer vesicles. Science.

[78] Otrin L., Marusic N., Bednarz C., Vidakovic-Koch T., Lieberwirt I., Landfester K., Sundmacher K. (2017). Toward Artificial Mitochondrion: Mimicking Oxidative Phosphorylation in Polymer and Hybrid Membranes. Nano Lett..

[79] Liu X.M., Zhou P., Huang Y.D., Li M., Huang X., Mann S. (2016). Hierarchical Proteinosomes for Programmed Release of Multiple Components. Angew. Chem. Int. Edit..

[80] Oberholzer T., Wick R., Luisi P.L., Biebricher C.K. (1995). Enzymatic RNA replication in self-reproducing vesicles: An approach to a minimal cell. Biochem. Biophys. Res. Commun..

[81] Wick R., Luisi P.L. (1996). Enzyme-containing liposomes can endogenously produce membrane-constituting lipids. Chem. Biol..

[82] Ota S., Yoshizawa S., Takeuchi S. (2009). Microfluidic formation of monodisperse, cell-sized, and unilamellar vesicles. Angew. Chem. Int. Ed. Engl..

[83] Glavas-Dodov M., Fredro-Kumbaradzi E., Goracinova K., Simonoska M., Calis S., Trajkovic-Jolevska S., Hincal A.A. (2005). The effects of lyophilization on the stability of liposomes containing 5-FU. Int. J. Pharm..

[84] Sun B., Chiu D.T. (2005). Determination of the encapsulation efficiency of individual vesicles using single-vesicle photolysis and confocal single-molecule detection. Anal. Chem..

[85] Tan Y.C., Hettiarachchi K., Siu M., Pan Y.R., Lee A.P. (2006). Controlled microfluidic encapsulation of cells, proteins, and microbeads in lipid vesicles. J. Am. Chem. Soc..

[86] Zhu P.A., Wang L.Q. (2017). Passive and active droplet generation with microfluidics: A review. Lab. Chip.

[87] Maamar H., Raj A., Dubnau D. (2007). Noise in gene expression determines cell fate in Bacillus subtilis. Science.

[88] Chang H.H., Hemberg M., Barahona M., Ingber D.E., Huang S. (2008). Transcriptome-wide noise controls lineage choice in mammalian progenitor cells. Nature.

[89] Graf T., Stadtfeld M. (2008). Heterogeneity of Embryonic and Adult Stem Cells. Cell Stem Cell.

[90] Delgado R.N., Lim D.A. (2015). Embryonic Nkx2.1-expressing neural precursor cells contribute to the regional heterogeneity of adult V-SVZ neural stem cells. Dev. Biol..

[91] Luisi P.L., Allegretti M., de Souza T.P., Steiniger F., Fahr A., Stano P. (2010). Spontaneous Protein Crowding in Liposomes: A New Vista for the Origin of Cellular Metabolism. ChemBioChem.

[92] De Souza C.A., Teixeira P.C., Faria R.X., Krylova O., Pohl P., Alves L.A. (2012). A consensus segment in the M2 domain of the hP2X(7) receptor shows ion channel activity in planar lipid bilayers and in biological membranes. Biochim. Biophys. Acta.

[93] Hansen M.M., Meijer L.H., Spruijt E., Maas R.J., Rosquelles M.V., Groen J., Heus H.A., Huck W.T. (2016). Macromolecular crowding creates heterogeneous environments of gene expression in picolitre droplets. Nat. Nanotechnol..

[94] Matsuura T., Tanimura N., Hosoda K., Yomo T., Shimizu Y. (2017). Reaction dynamics analysis of a reconstituted Escherichia coli protein translation system by computational modeling. Proc. Natl. Acad. Sci. USA.

[95] Chizzolini F., Forlin M., Martin N.Y., Berloffa G., Cecchi D., Mansy S.S. (2017). Cell-Free Translation Is More Variable than Transcription. ACS Synth. Biol..

[96] Elowitz M.B., Levine A.J., Siggia E.D., Swain P.S. (2002). Stochastic gene expression in a single cell. Science.

[97] Karig D.K., Jung S.Y., Srijanto B., Collier C.P., Simpson M.L. (2013). Probing Cell-Free Gene Expression Noise in Femtoliter Volumes. ACS Synth. Biol..

[98] Nishimura K., Tsuru S., Suzuki H., Yomo T. (2015). Stochasticity in Gene Expression in a Cell-Sized Compartment. ACS Synth. Biol..

[99] Stogbauer T., Windhager L., Zimmer R., Radler J.O. (2012). Experiment and mathematical modeling of gene expression dynamics in a cell-free system. Integr. Biol..

[100] Kempf N., Remes C., Ledesch R., Zuchner T., Hofig H., Ritter I., Katranidis A., Fitter J. (2017). A Novel Method to Evaluate Ribosomal Performance in Cell-Free Protein Synthesis Systems. Sci. Rep..

[101] Niess A., Failmezger J., Kuschel M., Siemann-Herzberg M., Takors R. (2017). Experimentally Validated Model Enables Debottlenecking of in Vitro Protein Synthesis and Identifies a Control Shift under in Vivo Conditions. ACS Synth. Biol..

[102] Schwander T., von Borzyskowski L.S., Burgener S., Cortina N.S., Erb T.J. (2016). A synthetic pathway for the fixation of carbon dioxide in vitro. Science.

[103] Bhattacharya A., Brea R.J., Niederholtmeyer H., Devaraj N.K. (2019). A minimal biochemical route towards de novo formation of synthetic phospholipid membranes. Nat. Commun..

[104] Van Nies P., Westerlaken I., Blanken D., Salas M., Mencia M., Danelon C. (2018). Self-replication of DNA by its encoded proteins in liposome-based synthetic cells. Nat. Commun..

